# Synbiotics Easing Renal Failure by Improving Gut Microbiology II (SYNERGY II): A Feasibility Randomized Controlled Trial

**DOI:** 10.3390/nu13124481

**Published:** 2021-12-15

**Authors:** Catherine McFarlane, Rathika Krishnasamy, Tony Stanton, Emma Savill, Matthew Snelson, Gabor Mihala, Jaimon T. Kelly, Mark Morrison, David W. Johnson, Katrina L. Campbell

**Affiliations:** 1 School of Medicine, University of Queensland, Brisbane, QLD 4006, Australia; 2 Sunshine Coast University Hospital, Birtinya, QLD 4575, Australia; rathika.krishnasamy@health.qld.gov.au (R.K.); tony.stanton@health.qld.gov.au (T.S.); emma.savill@health.qld.gov.au (E.S.); 3 Australasian Kidney Trials Network, University of Queensland, Brisbane, QLD 4102, Australia; david.johnson2@health.qld.gov.au; 4 School of Medicine, Griffith University, Birtinya, QLD 4575, Australia; 5 Department of Diabetes, Monash University, Melbourne, VIC 3004, Australia; matthew.snelson@monash.edu; 6 School of Medicine and Dentistry, Griffith University, Brisbane, QLD 4111, Australia; g.mihala@griffith.edu.au; 7 Centre for Applied Health Economics, Griffith University, Brisbane, QLD 4111, Australia; 8 Menzies Health Institute Queensland, Griffith University, Brisbane, QLD 4111, Australia; jaimon.kelly@uq.edu.au (J.T.K.); katrina.campbell@health.qld.gov.au (K.L.C.); 9 Diamantina Institute, Faculty of Medicine, University of Queensland, Brisbane, QLD 4102, Australia; m.morrison1@uq.edu.au; 10 Department of Nephrology, Princess Alexandra Hospital, Brisbane, QLD 4102, Australia; 11 Healthcare Excellence and Innovation, Metro North Hospital and Health Service, Brisbane, QLD 4029, Australia

**Keywords:** synbiotic, gastrointestinal microbiome, kidney disease, randomized controlled trial, *p*-cresyl sulphate, indoxyl sulfate

## Abstract

Synbiotics have emerged as a therapeutic strategy for modulating the gut microbiome and targeting novel cardiovascular risk factors, including uremic toxins indoxyl sulfate (IS) and *p*-cresyl sulfate (PCS). This study aims to evaluate the feasibility of a trial of long-term synbiotic supplementation in adults with stage 3–4 chronic kidney disease (CKD). Adult participants with CKD and estimated glomerular filtration rate (eGFR) of 15–60 mL/min/1.73 m^2^) were recruited between April 2017 and August 2018 to a feasibility, double-blind, placebo-controlled, randomized trial of synbiotic therapy or matched identical placebo for 12 months. The primary outcomes were recruitment and retention rates as well as acceptability of the intervention. Secondary outcomes were treatment adherence and dietary intake. Exploratory outcomes were evaluation of the cardiovascular structure and function, serum IS and PCS, stool microbiota profile, kidney function, blood pressure, and lipid profile. Of 166 potentially eligible patients, 68 (41%) were recruited into the trial (synbiotic *n* = 35, placebo *n* = 33). Synbiotic and placebo groups had acceptable and comparable 12-month retention rates (80% versus 85%, respectively, *p* = 0.60). Synbiotic supplementation altered the stool microbiome with an enrichment of *Bifidobacterium* and *Blautia* spp., resulting in a 3.14 mL/min/1.73 m^2^ (95% confidence interval (CI), −6.23 to −0.06 mL/min/1.73 m^2^, *p* < 0.01) reduction in eGFR and a 20.8 µmol/L (95% CI, 2.97 to 38.5 µmol/L, *p* < 0.01) increase in serum creatinine concentration. No between-group differences were observed in any of the other secondary or exploratory outcomes. Long-term synbiotic supplementation was feasible and acceptable to patients with CKD, and it modified the gastrointestinal microbiome. However, the reduction in kidney function with synbiotics warrants further investigation.

## 1. Introduction

Individuals with chronic kidney disease (CKD) have a significantly increased risk of cardiovascular disease (CVD) and are more likely to die from cardiovascular-related complications than progress to kidney replacement therapy [1]. Framingham risk factors only partially explain this elevated cardiovascular risk [2], leading many researchers to explore novel risk factors such as uremic toxins. The uremic toxins, indoxyl sulfate (IS) and *p*-cresyl sulfate (PCS), have increasingly been associated with accelerated kidney disease progression and cardiovascular morbidity and mortality [3,4,5]. These toxins are produced through the metabolism of the dietary amino acids, tryptophan (IS) and tyrosine (PCS), respectively, by the gut microbiota [6]. Both IS and PCS are predominantly bound to plasma proteins, such as albumin, and rely on kidney excretion. An altered gut microbiota and reduced kidney clearance are both contributing factors to increased serum toxin concentrations in CKD [7].

The altered gut microbiota seen in patients with CKD may be therapeutically modifiable. Synbiotic supplementation (the co-administration of prebiotics and probiotics) has emerged over the last decade as a reportedly innocuous intervention targeting the microbial generation of IS and PCS. The key hypothesis is that controlling toxin generation by competitively decreasing protein-fermenting intestinal microbiota can slow CKD progression and reduce associated cardiovascular complications. The Synbiotics Easing Renal Failure by Improving Gut Microbiology (SYNERGY) study [8], a proof-of-concept study, determined that synbiotic supplementation reduced PCS and favorably altered the gastrointestinal microbiome. However, to date, studies of synbiotic interventions in CKD have been limited, hampered by uncertain or high risk of bias and conflicting findings [9]. Whilst these interventions report high levels of adherence and minimal side effects, the duration of supplementation was short, ranging from 1 to 2 months [9]. Dietary interventions are exceptionally challenging with up to 60% of participants being unable to adhere to recommended dietary changes long-term (1 year) [10]. Meanwhile, medication non-adherence in chronic conditions is common, with rates averaging 50% and higher [11]. Further, adherence to nutrition supplementation in the chronic disease population remains largely unexplored. Accordingly, this study aimed to evaluate the feasibility and acceptability of long-term synbiotic supplementation in people with stage 3–4 CKD. In addition, this study explored the effect of synbiotic supplementation on markers of cardiovascular risk, microbial-derived uremic toxins, and gastrointestinal (stool) microbiota.

## 2. Materials and Methods

The Synbiotics Easing Renal Failure by Improving Gut Microbiology II (SYNERGY II) study was a double-blind, placebo-controlled, randomized controlled trial assessing the feasibility and acceptability of 12-month synbiotic supplementation in patients with moderate to severe CKD. Ethical approval was granted through the Metro South Human Research Ethics Committee (HREC/16/QPAH/336) and the University of Queensland Human Research Ethics committee, and the study adhered to the Declaration of Helsinki. All patients provided written consent prior to enrolment and participation. The SYNERGY II study was registered with the Australian and New Zealand Clinical Trials Registry (ACTRN12617000324314).

### 2.1. Participants

Patients from two tertiary kidney care outpatient departments with an estimated glomerular filtration rate (eGFR) between 15 and 60 mL/min/1.73 m^2^, aged ≥ 18 years of age were eligible for inclusion. Exclusion criteria were as follows: anticipated dialysis commencement within 12 months or anticipated death within 6 months; non-English speaking or unable to give informed consent; a clinically significant change in immunosuppressant dose within 6 months; receiving or had received radiation to the bowel or had a large bowel resection; consumed pre-, probiotic, or antibiotic therapy within 1 month of study commencement; medically diagnosed and active irritable bowel syndrome, Crohn’s disease, or ulcerative colitis; cirrhotic liver disease; or severely malnourished (Subjective Global Assessment: C).

A local site investigator or research nurse utilized daily outpatient appointment lists and relevant hospital databases to screen potential participants for eligibility. Participants were approached and invited to participate following discussion with their treating nephrologist.

### 2.2. Study Design

At the first study visit, participants were randomly assigned to either synbiotic supplements or placebo for 12 months. The randomization was completed by a computer-generated random number (1:1 ratio, blocks of 4, stratified by CKD stage and presence of diabetes), which was carried out by an independent investigator not involved in the recruitment or implementation of the study protocol. All participants received face-to-face or telephone dietary education and counseling with a qualified dietitian in line with evidence-based guidelines [12,13] at their commencement in the trial.

### 2.3. Intervention

The prebiotic component of the synbiotic therapy consisted of 20 g/day of high-resistant starch fiber supplement (Hi-Maize 260, 50% resistant starch; Ingredion), and the probiotic component provided 4.5 × 10^11^ colony-forming units (CFU)/day of nine different strains from three different genera (*Bifidobacteria*, *Lactobacillus*, and *Streptococcus*; Swiss Mendes). The placebo group received waxy maize powder (Ingredion) and maltodextrin (Swiss Mendes) in lieu of the prebiotic and probiotic, respectively. Both prebiotics and probiotics were packaged offsite and labeled by an independent investigator. The prebiotic component in this present study differed from that used in the SYNERGY study [8], which used 15 g/day across fructooligosaccharide (FOS), galactooligosaccharide (GOS), and inulin. Short-chain carbohydrates, such as FOS and GOS, are rapidly fermented in the proximal colon, while resistant starch is slowly fermented and can reach the distal parts of the colon, yielding a greater production of short-chain fatty acids (SCFA) [14]. The probiotic component in this present study had identical taxa to that used in the SYNERGY study [8]; however, it had a higher CFU. The probiotic contained strains from the *Lactobacillus* (*L. acidophilus*, *L. plantarum*, *L. paracasei*, *L. delbrueckii* subsp. *Bulgaricus*), *Bifidobacteria* (*B. breve, B. longum*, *B. infantis*) and *Streptococcus* (*S. thermophilus*) genera, which have limited enzymatic capacity to produce uremic toxins and competitively decrease bacteria that do [15,16].

**Figure 1:**
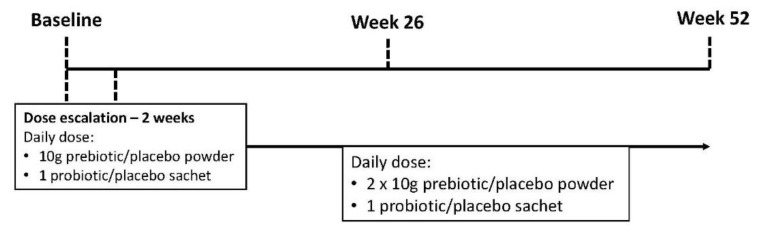
The Synbiotics Easing Renal Failure by Improving Gut Microbiology II (SYNERGY II) study schema.

The study design (Figure 1) included a dose escalation to avoid potential adverse gastrointestinal symptoms. In both arms, the prebiotic/placebo were commenced at a one-half dose for the first 2 weeks, consisting of 10 g (one level scoop) of the prebiotic/placebo powder and one probiotic/placebo sachet. This was taken in the morning with food. Following dose escalation, participants included an additional prebiotic/placebo dose (10 g powder) with their evening meal. A qualified dietitian provided telephone consultations fortnightly for the first 2 months and then monthly for the remainder of the intervention to verify adherence and assess adverse events. If the participant experienced any adverse gastrointestinal symptoms associated with the supplement, the supplement dose was modified. Throughout the intervention, participants were provided individualized and tailored dietary advice in line with best practice guidelines and were encouraged to maintain a stable dietary intake, focusing on not altering protein and fiber intake.

### 2.4. Outcome Measures

#### Primary Outcome

The primary outcome was the feasibility and acceptability of long-term synbiotic supplementation. Feasibility was measured through recruitment and retention rate. Recruitment rate was collected and stored in a password-protected Microsoft Excel database throughout the trial. Retention rate was measured in both groups at 3, 6, 9, and 12 months. Successful retention was defined as 80% at the 12-month study end. Acceptability was measured through gastrointestinal symptoms, stool frequency, and stool consistency. Gastrointestinal symptoms, stool frequency, and stool consistency were measured at baseline, 3, 6, 9, and 12 months. Gastrointestinal symptoms were measured using the Gastrointestinal Symptoms Rating Scale (GSRS) [17], which is a 15-item survey that combines symptoms into 5 clusters: reflux, abdominal pain, indigestion, diarrhea, and constipation. The GSRS has a 7-point Likert scale in which the intensity of each symptom is graded from 1 to 7, with a higher score indicating more severe symptoms. Stool frequency was measured by a single question asking the participant the number of times their bowels had opened in the previous 24 h period. The Bristol Stool Score (BSS) [18], a 7-point scale, was used to classify stool consistency.

### 2.5. Secondary Outcomes

Secondary outcomes were treatment adherence and dietary intake. Adherence was measured by sachet count and powder weight, and it was defined as consuming 80% or more of the prescribed pre- and probiotics. Participants’ dietary intakes were assessed using a 7-day food record at baseline and at the final study visit by the same dietitian. Dietary data were analyzed using Food Works 9 (Xyris Software; Version 9.0.3932). All adverse and serious adverse events (SAE) were documented and reported to the ethics committee for review. The validated ‘Assessment of Quality of Life’ questionnaire (AQoL-4D), a 12-item instrument, was used to assess health-related quality of life [19]. The AQoL-4D instrument measures health-related quality of life across 4 measures: independent living, social relationships, senses, and mental health. The AQoL-4D uses a 4-point Likert scale in which each response is graded from 1 (a ‘good’ health state) to 4 (the ‘worst’ health state).

### 2.6. Exploratory Outcomes

Exploratory outcome measures included echocardiographic measurements of global longitudinal strain (GLS), left ventricular mass index (LVMI) and ejection fraction, serum creatinine, eGFR, free and protein-bound serum concentrations of serum IS and PCS, and stool microbiota analysis (opt-in). Outcome measures were collected at baseline and 12 months and were analyzed using validated methods detailed in Appendix A. The DNA extraction and sequencing methods are described in detail in Appendix A.

### 2.7. Sample Size

The sample size of 68 participants to be randomized was based on a recruitment rate of 20% of eligible patients to within a 95% confidence interval of ±10%. The recruitment rate of 20% is based on the previous SYNERGY trial [8].

### 2.8. Statistical Analysis

Summary statistics for patients’ characteristics were expressed as means ± standard deviations for normally distributed continuous data, medians (interquartile ranges) for continuous non-normally distributed data, and frequencies (percentages) for categorical data. Baseline differences between intervention and placebo groups were evaluated by chi-square or Fisher’s exact tests for categorical variables and Student’s *t*-test or Mann–Whitney test, for normal or skewed continuous variables, respectively.

Recruitment, retention, and adherence rates were estimated using simple descriptive statistics (counts and percentages). To determine the difference in the GSRS and BSS between the two study groups, the chi-square test was used. The secondary outcome analyses were undertaken using the analysis of covariance regression method, i.e., adjusted for the baseline measure of the variable. The outcome values were transformed where not normally distributed. All analyses followed an intention-to-treat principle; however, a sensitivity analysis was performed to evaluate additional effects of eGFR, dietary protein/fiber ratio, and antibiotics. The analysis was conducted in Stata (Version 15; StataCorp., College Station, TX, USA) with the significance level set at 0.05.

The stool microbiota between the synbiotic and placebo groups were compared as described by McFarlane et al. [20]. Briefly, the richness and Shannon index values were compared using mixed effect linear models. Data/sample clustering was examined using both unsupervised analysis (Principal Coordinates Analysis (PCoA) of Bray–Curtis dissimilarity metrics) and supervised analyses (redundancy analysis (RDA)) constrained by the synbiotic or placebo intervention. These data were also subjected to centered-log ratio transformation to support multivariate analyses by sparse Partial Least Squares Discriminant Analysis (sPLS-DA) [21]. The sPLS-DA function of the Mixomics micMC package was used to identify discriminatory taxa [22]. Those bacterial taxa considered different between the two treatment arms were further tested for significance and after adjustment for multiplicity, as described by McFarlane et al. [20] and were considered statistically significant if both the *P* and FDR values were less than 0.05.

## 3. Results

Participants were recruited from two kidney care outpatient departments from April 2017 to August 2018 and randomized to synbiotic or placebo group. Of the 68 participants who completed their baseline visit, 66% were men, had a median age of 70 years (IQR 58–75 years), and had a median eGFR of 34.0 mL/min/1.73 m^2^ (IQR 27.0–41.0 mL/min/1.73 m^2^) (Table 1). A total of 56 participants completed the 12-month intervention with 40 participants completing the optional fecal sub-study. There were no statistically significant differences in the demographic, clinical, dietary, and biochemical parameters between groups at baseline.

### 3.1. Feasibility

#### 3.1.1. Recruitment

**Figure 2:**
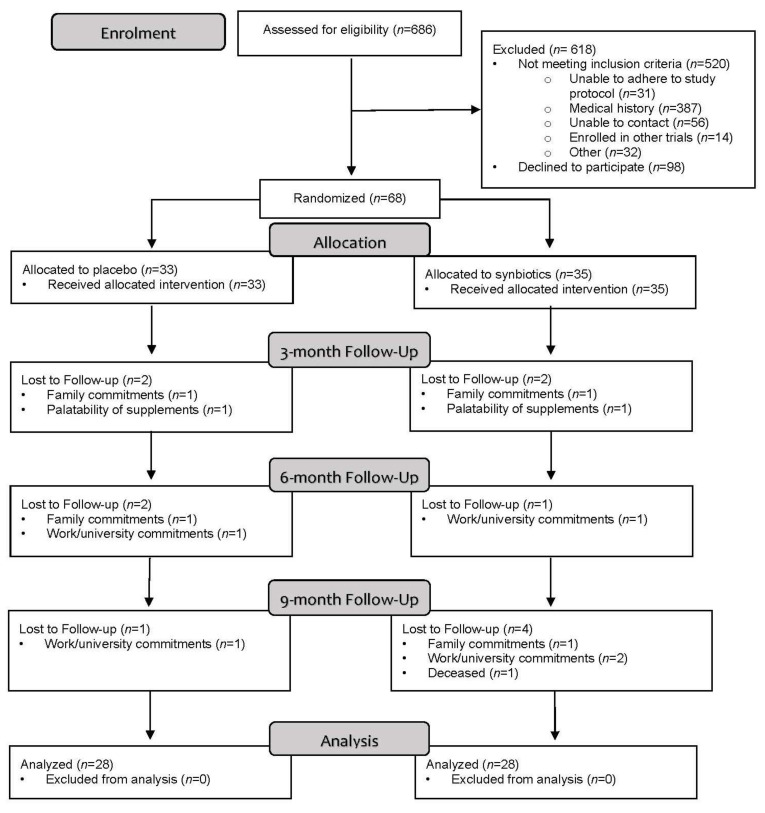
CONSORT flow diagram.

The flow of participants through the SYNERGY II study is described in the CONSORT diagram (Figure 2). A total of 686 individuals were screened, of whom 520 (76%) were not eligible, 98 (14%) declined to participate, and 68 (10%) were recruited into the study. Of the eligible participants, 41% (68/166) consented to participate in the SYNERGY II trial. Of the 98 individuals who declined to participate, ‘not interested’ was the most common reason for not participating (82, 84%), which was followed by travel burden (8, 9%) and family commitments (6, 7%).

#### 3.1.2. Retention

The retention rate was 82%, with 56 of the randomly allocated participants completing the 12-month intervention. Twelve (18%) participants withdrew from the study with no difference in withdrawals between groups (synbiotic: 7, placebo: 5; *p* = 0.60). Work or university commitments were the main reason for withdrawal (5, 42%) followed by family commitments or caregiver responsibilities (4, 33%). One participant from each group withdrew at week 2 and cited the palatability of the study products as the reason for withdrawal. The majority of withdrawals occurred at ≥6 months (8, 67%).

### 3.2. Acceptability

Table 2 shows changes in the acceptability outcomes between the two groups. There were no significant changes in gastrointestinal symptoms or stool consistency between the two groups.

### 3.3. Secondary Outcomes

Overall, adherence was excellent in both placebo and synbiotic groups (median intake 92% (86–95%) versus 90% (84–95%); *p* = 0.40, respectively). There was no difference in adherence to prebiotics (median intake 92% (83–98) placebo versus 89% (83–92) synbiotic group; *p* = 0.40) or probiotics (median intake 91% (88–98) placebo versus 90% (84–95) synbiotic group; *p* = 0.60) between the groups. There were no significant changes in dietary intakes of energy, protein, fiber, and protein/fiber ratio between the groups (Table 2). There were 18 serious adverse events (SAE) in total: seven occurred in the placebo group, and 11 occurred in the synbiotic group. Initial hospitalization accounted for 17 SAEs. One participant died during the study period for reasons unrelated to the study (Table 3).

### 3.4. Exploratory Outcomes

Table 4 shows changes in exploratory outcomes between groups. No differences in cardiac measures of GLS, LVMI, or ejection fraction were observed between groups. Furthermore, no differences were observed between free and total uremic toxins between placebo and synbiotic groups. Synbiotic supplementation resulted in a 3.14 mL/min/1.73 m^2^ (95% CI, −6.23 to −0.06 mL/min/1.73 m^2^, *p* < 0.01) reduction in eGFR and a 20.8 µmol/L (95% CI, 2.97 to 38.5 µmol/L, *p* < 0.01) increase in serum creatinine concentration. Two (6%) participants in the synbiotic group had a >40% decline in eGFR over 12 months. Additional adjustments for baseline eGFR, dietary protein/fiber ratio, and antibiotics did not change the results (data not shown).

### 3.5. Gastrointestinal (Stool) Microbiota

**Figure 3:**
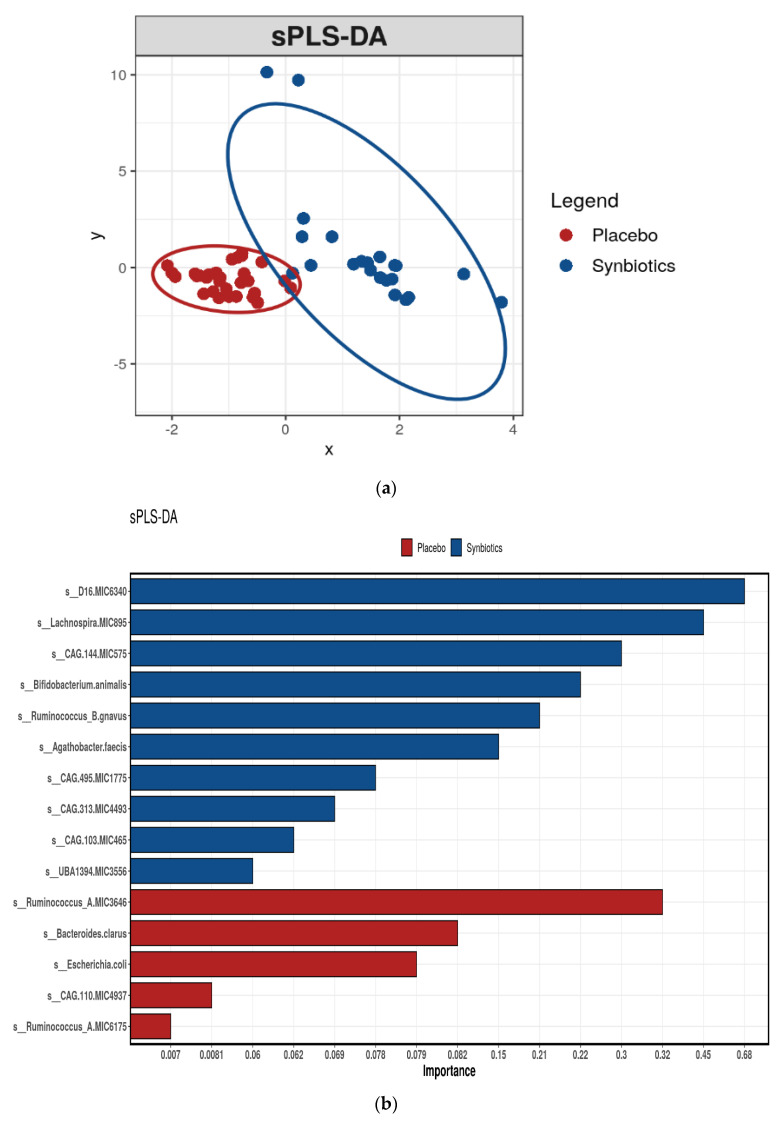
(**a**) sPLS-DA of gut microbiome composition by intervention. (**b**) Species differentiating between gut microbiota profiles of participants after placebo or synbiotic supplementation as identified by sparse Partial Least Squares Discriminant Analysis (sPLS-DA).

The coverage of the microbiota diversity for all samples was high with a rarefied sequencing depth of 4 million reads with 298 species identified. No differences were observed in Richness and Shannon’s index between synbiotic or placebo groups (Supplemental Figure S1A,B). While an unsupervised analysis (PCoA) revealed no discernible clustering, a supervised analysis (sPLS-DA) at the species level identified differential taxa between groups (Figure 3a). Specifically, unclassified members of *Oscillospiraceae* and *Coprobacteraceae* families, *Lachnospira* genus, *Bifidobacterium animalis*, and *Ruminococcus B gnavus* were discriminatory taxa for the synbiotic group (Figure 3b), while unclassified members of *Ruminococcus A*, *Bacteroides clarus*, and *Escherichia coli* were taxa that were discriminatory for the placebo group. The most noticeable effect of synbiotic therapy was a 2.4-fold increased relative abundance of *Bifidobacterium animalis* (*p* < 0.001, FDR = 0.04, Supplemental Table S1) and unclassified *Blautia* spp. (*p* = 0.004, FDR = 0.47). There were concordant 1.7-fold decreases in *Bacteroides cellulosilyticus* and an unclassified *Ruminiclostridium* spp. (both *p* < 0.05, FDR = 0.47), although significance did not hold once adjusted for multiple comparisons. The RDA demonstrated that 17% of the variance in gastrointestinal microbial composition could be explained by synbiotic supplementation (F = 1.43, *p* = 0.001, Supplemental Figure S2).

#### Functional Assessment of the Gastrointestinal Microbiome

Supervised analysis (sPLS-DA) showed that the tricarboxylic acid (TCA) cycle, carboxylate degradation, acetyl coenzyme A (CoA) biosynthesis, and amino acid degradation were the most discriminating microbial function after synbiotic supplementation, while protein modification was the most discriminating microbial function after placebo (Supplemental Figure S3). The most pronounced effect observed with synbiotic therapy was a 1.8-fold increase (*p* < 0.001, FDR = 0.043) in the microbial function methane oxidation to methanol I pathway.

## 4. Discussion

This study is the first to examine the feasibility of long-term synbiotic supplementation in patients with CKD. There was a high rate of recruitment and retention, with 41% of eligible people consenting to be involved in the study and 82% of participants completing the study. Furthermore, synbiotic therapy achieved excellent compliance and did not result in altered gastrointestinal symptoms or stool consistency. Together, these findings indicate a good uptake and acceptance of synbiotic supplementation in people with CKD.

Overall, there has been limited evidence on the feasibility and acceptability of synbiotic supplementation in CKD or other chronic conditions. The rate of attrition (18%) in SYNERGY II was comparable to that in other synbiotic interventions [8,23] with durations of 1–3 months, which further strengthens feasibility given that the duration of SYNERGY II was 12 months. In this present study, synbiotic therapy was deemed acceptable by study participants with a high level of adherence and tolerance to treatment. Similar findings have been reported in other synbiotic interventions with adherence ranging from 82 to 94% [8,24,25], with minimal gastrointestinal side effects reported [26].

The present study observed no effect on echocardiographic parameters, blood pressure, and lipid profile. This is in agreement with recent meta-analyses that highlighted that supplementation had no effect on serum lipids [9,27]. To our knowledge, no prior intervention study has reported data on GLS, LVMI, and blood pressure after synbiotic supplementation.

A novel and unexpected finding of the SYNERGY II trial was that participants in the synbiotic group experienced a significant decrease in our exploratory outcomes eGFR and increase in serum creatinine compared with the placebo group. It is prudent to note that this was an underpowered exploratory outcome, and the observed difference may have been due to chance with the numeric increase in eGFR in the placebo group. However, this finding is in contrast to the findings of previous short-term studies that have shown synbiotic supplementation to have no effect on eGFR [8,23] or serum creatinine concentration [23]. In the SYNERGY trial [8], a proof-of-concept study, the daily use of prebiotics (15 g/day across fructooligosaccharide, galactooligosaccharide, inulin) and probiotics (4.5 × 10^10^ CFU/day (nine strains across *Lactobacillus*, *Bifidobacteria*, *Streptococcus*)) for 6 weeks did not change eGFR. However, a significant increase in albuminuria, a marker of kidney damage, was reported. SYNERGY II, a pragmatically designed feasibility study, did not routinely collect 24 h urine samples, precluding measurement of albuminuria and proteinuria. Similarly, Dehghani et al. [23] reported no change in eGFR or serum creatinine after 6 weeks of supplementation with prebiotic (fructooligosaccharide) and probiotic (seven strains across *Lactobacillus*, *Bifidobacteria*, *Streptococcus*). A systematic review and meta-analysis of 16 studies of pre-, pro-, and synbiotic supplementation involving 645 adult participants [9] found that nutritional supplementation probably made little or no difference to kidney function, as measured by eGFR (three studies, 132 participants, mean difference (MD) 0.34 mL/min/1.73 m^2^, *p* = 0.79, I^2^ = 0%), although the certainty of the evidence was limited by imprecision and risk of bias. Conversely, a meta-analysis of 13 studies involving 721 participants [28] reported that the consumption of prebiotics, probiotics, and synbiotics resulted in a non-significant reduction in eGFR (six studies, 376 participants, MD −2.00 mL/min/1.73 m^2^, 95% CI −5.15 to 1.16, *p* = 0.22, I^2^ = 89%). These findings were again limited by risk of bias, imprecision, and high heterogeneity. Therefore, the effects of pre-, pro-, and synbiotics on kidney function remain uncertain. Whether pre-, pro-, and synbiotics (or at least certain types of these agents) may have a deleterious effect on kidney function requires further and more rigorous investigation.

Our study also provides new insights into the effect of synbiotic therapy on gastrointestinal microbiota in CKD. Increases were observed in bacterial taxa that are well-recognized for their specialized capabilities in terms of acetate (e.g., *Bifidobacterium* spp.) [29] and butyrate formation (e.g., *Blautia* spp.) [30]. This is also reflected in the functional categories, which showed that the TCA cycle and acetyl CoA biosynthesis were discriminant after synbiotic supplementation. Acetyl CoA biosynthesis is a key enzymatic step in the formation of butyrate and acetate [31,32]. Both acetate and butyrate are a desirable outcome of gut fermentation, as they have been associated with improved integrity of the intestinal epithelium and enhanced anti-inflammatory effects [33]. The shift in microbial composition after synbiotic supplementation in this present study is consistent with two other synbiotic studies that used combinations of *Lactobacillus*, *Bifidobacterium*, and *Streptococcus* spp. with inulin, fructo-oligosaccharides (FOS), and galacto-oligosaccharides (GOS) [8], and *Lactobacillus* and *Bifidobacterium* spp. and inulin [34]. More detailed metabolomic and microbiome studies are required to further understand the effect of symbiotic therapy.

In this study, 12 months of synbiotic supplementation was found to have no effect on serum concentrations of the uremic toxins, IS and PCS. This is supported by a recent meta-analysis that reported no effect on IS and PCS after supplementation [9]. However, this meta-analysis was limited by suboptimal study quality and heterogeneity inclusive of study duration and pre- and probiotic formulations. To date, there have been five other synbiotic intervention studies: three controlled trials in stage 3 and 4 CKD [8,23,35] and two controlled trials in patients on hemodialysis [26,34]. The duration of supplementation ranged from four to eight weeks. Of these, two measured uremic toxins [8,35]. In contrast to our findings, the SYNERGY trial [8] reported a mean 14 μmol/L reduction in serum PCS after six weeks of prebiotics (15 g/day across fructooligosaccharide, galactooligosaccharide, inulin) and probiotics (4.5 × 10^10^ CFU/day (nine strains across *Lactobacillus*, *Bifidobacteria*, *Streptococcus*)) supplementation. Similarly, Guida et al. [35] determined that four weeks of prebiotic (6.6 g/day of inulin) and probiotic (5.7 × 10^10^ CFU/day (nine strains across *Lactobacillus*, *Bifidobacteria*, *Streptococcus*)) supplementation reduced plasma p-cresol, which is a precursor of PCS. Therefore, it is plausible that the prebiotic and probiotic formulation, dose, and duration of treatment are critical to inducing changes in uremic toxin concentrations.

The major strengths of the SYNERGY II study include its robust design (randomized, double-blind, placebo-controlled) and the provision of both taxonomic and functional understanding of the gastrointestinal microbiome after synbiotic supplementation. To our knowledge, the SYNERGY II study is the longest synbiotic intervention and the first to explore the feasibility of synbiotic supplementation in the CKD population. Potential dietary confounders were controlled for by using validated dietary assessment methods by a qualified dietitian who was blinded along with participants to the intervention. However, balanced against these strengths, the SYNERGY II feasibility study was limited by a small sample size that has limited statistical power to detect significant changes in secondary clinical outcomes (cardiovascular risk markers and uremic toxins) and use of surrogate outcome measures (uremic toxins and stool microbiome). Furthermore, our study population was limited to participants with an eGFR between 15 and 60 mL/min/1.73 m^2^, which may have limited the generalizability of our findings to other CKD patients. Finally, metabolomic analysis would be useful in future studies to provide further insight into the metabolite performance of the microbiome.

In summary, this study indicates that long-term synbiotic supplementation is feasible and acceptable for adults with stage 3 to 4 CKD. There were notable alterations in the gastrointestinal microbiome and some functional groups involved in the gastrointestinal microbiota after synbiotic supplementation. However, the reduction in kidney function observed with synbiotic supplementation warrants further investigation.

## Figures and Tables

**Table 1:** Baseline characteristics of participants in the SYNERGY II study.

	Placebo (*n* = 33)	Synbiotics (*n* = 35)	Total (*n* = 68)
Sex (male)	22 (67%)	23 (66%)	45 (66%)
Age (years)	69 (56–73)	72 (66–76)	70 (58–75)
Weight (kg)	95.4 (78.6–119)	87.2 (7.7–95.2)	87.9 (74.0–102)
BMI (*n* = 67)	30.3 (11.3)	28.0 (8.9)	29.2 (25.2–36.3)
Cause of chronic kidney disease:			
diabetes	5 (15%)	10 (29%)	15 (22%)
hypertension/vascular	9 (27%)	5 (14%)	14 (21%)
glomerulonephritis	5 (15%)	8 (23%)	13 (19%)
other	14 (42%)	12 (34%)	26 (38%)
Comorbidities (treated):			
hypertension	27 (82%)	25 (71%)	52 (76%)
diabetes	14 (42%)	16 (46%)	30 (44%)
hyperlipidemia	11 (33%)	8 (23%)	19 (28%)
cardiovascular disease	10 (30%)	16 (46%)	26 (28%)
Medications:			
ACE inhibitor	14 (42%)	14 (40%)	28 (41%)
angiotensin II receptor blocker	15 (45%)	14 (40%)	29 (43%)
diuretic medication	13(39%)	15 (43%)	28 (41%)
Number of antihypertensive medications	2.4 ± 1.3	2.4 ± 1.7	2.4 ± 1.5
Antibiotics	1 (3%)	2 (6%)	3 (4%)
Echocardiographic characteristics			
Global longitudinal strain (%) (*n* = 66)	−18.0 ± 2.7	−17.2 ± 3.4	−17.6 ± 3.1
Left ventricular mass index (g/m^2^) (*n* = 67)	82.9 (72.5–96.0)	84.6 (64.0–105)	83.1 (65.0–99.0)
Ejection fraction (%) (*n* = 66)	61.0 (56.0–65.0)	63.0 (58.0–68.0)	61.5 (57.0–66.0)
eGFR (mL/min/1.73 m^2^)	36.0 (29.0–44.0)	31.5 (26.0–37.0)	34.0 (27.0–41.0)
CKD stage 3 (*n* = 41)	42.7 (36.0–47.0)	38.5 (34.0–43.0)	40.7 (35.0–44.0)
CKD stage 4 (*n* = 27)	24.4 (19.0–29.0)	23.6 (20.0–27.0)	23.9 (19.5–28.5)
Creatinine (µmol/L)	163 (131–191)	168 (135–217)	168 (133–198)
Cholesterol (mmol/L)	4.20 (3.60–5.20)	4.30 (3.70–4.80)	4.30 (3.60–5.00)
HDL cholesterol (mmol/L)	1.10 (0.90–1.20)	1.10 (0.90–1.30)	1.10 (0.90–1.30)
LDL cholesterol (mmol/L)	2.40 (2.00–2.95)	2.35 (1.85–2.75)	2.40 (1.90–2.80)
Triglycerides (mmol/L)	1.80 (1.50–2.40)	1.70 (1.05–2.35)	1.80 (1.10–2.40)
Uremic toxins (*n* = 65)			
Total indoxyl sulfate (µmol/L)	16.0 (9.0–26.0)	16.5 (9.0–26.0)	16.0 (9.0–26.0)
Total p-cresyl sulfate (µmol/L)	76.0 (30.0–153)	83.0 (37.5–115)	82.0 (31.0–129)
Free indoxyl sulfate (µmol/L)	0.80 (0.50–1.10)	0.65 (0.50–1.15)	0.80 (0.50–1.10)
Free p-cresyl sulfate (µmol/L)	2.80 (0.80–4.70)	2.25 (0.85–4.30)	2.30 (0.80–4.70)
Blood pressure			
Systolic BP (mmHg)	137 ± 21	141 ± 24	139 ± 23
Diastolic BP (mmHg)	79 ± 9	77 ± 8	78 ± 8
Dietary intake (*n* = 66)			
Energy (kJ/day)	7868 ± 1894	7281 ± 2125	7566 ± 2023
Energy (kJ/kg)	81 ± 26	90± 31	85 ± 29
Protein (g/day)	97 ± 27	85 ± 27	90 ± 28
Protein (g/kg)	1.0 ± 0.3	1.1 ± 0.5	1.0 ± 0.4
Fiber (g/day)	22 ± 7	21 ± 8	22 ± 8
Protein/fiber ratio	4.6 ± 1.5	4.4 ± 1.7	4.5 ± 1.6
GSRS score			
Mean score	1.5 ± 0.5	1.5 ± 0.5	1.5 ± 0.5
Score > 3	0 (0%)	1 (3%)	1 (1%)
Reflux	1.3 ± 0.5	1.4 ± 0.9	1.3 ± 0.7
Abdominal pain	1.4 ± 0.5	1.6 ± 0.8	1.5 ± 0.7
Indigestion	1.6 ± 0.6	1.6 ± 0.6	1.6 ± 0.6
Constipation	1.7 ± 1.2	1.4 ± 0.8	1.6 ± 1.0
Diarrhea	1.2 ± 0.4	1.3 ± 0.6	1.3 ± 0.5
Bristol stool score (1 to 7)	3.6 ± 1.3	3.7 ± 1.2	3.6 ± 1.2
No. of bowel motions in previous 24 h	1.3 ± 0.6	1.4 ± 0.7	1.3 ± 0.6
Patient-reported health score (AQoL-4D)	15.0 (13.0–17.0)	16.0 (13.0–18.0)	16.0 (13.0–17.5)

Data are presented as means ± SD, median (25th–75th percentiles), or frequency (%). ACE inhibitor, angiotensin-converting enzyme inhibitor; BMI, body mass index; BP, blood pressure; eGFR, estimated glomerular filtration rate; GSRS, Gastrointestinal Symptom Rating Scale; score > 3 indicates moderate discomfort.

**Table 2:** Dietary intake and gastrointestinal symptoms at the end of intervention and changes from baseline.

	Placebo (*n* = 28)	Change (95% CI) Placebo	Synbiotics (*n* = 28)	Change (95% CI) Synbiotics	*p*-Value ^a^
Dietary intake					
Energy (kJ/day)	8476 ± 2443	415 (−457–1287)	7316 ± 2086	−51.7 (−852–749)	0.21
Protein (g/day)	106 ± 37	9.11 (−4.98–23.2)	90 ± 31	2.64 (−10.7–16.0)	0.18
Fiber (g/day)	25 ± 8	1.14 (−1.59–3.88)	21 ± 8	−0.86 (−4.58–2.87)	0.06
Protein/fiber ratio	4.4 ± 1.5	0.08 (−0.51–0.68)	4.7 ± 1.7	0.27 (−0.60–1.15)	0.64
GSRS score					
Mean score	1.4 ± 0.4	−0.13 (−0.32–0.07)	1.4 ± 0.6	0.01 (−0.15–0.17)	0.93
Score > 3	0 (0%)		1 (4%)		
Reflux	1.3 ± 0.4	−0.09 (−0.31–0.13)	1.4 ± 0.8	0.11 (−0.24–0.45)	0.96
Abdominal	1.3 ± 0.4	−0.17 (−0.40–0.07)	1.2 ± 0.4	−0.23 (−0.45–0.00)	0.56
Indigestion	1.6 ± 0.5	−0.08 (−0.32–0.16)	1.7 ± 0.9	0.08 (−0.16–0.32)	0.58
Constipation	1.5 ± 0.8	−0.27 (−0.67–0.12)	1.4 ± 1.0	0.06 (−0.26–0.37)	0.75
Diarrhea	1.2 ± 0.4	−0.04 (−0.22–0.15)	1.3 ± 0.5	0.02 (−0.20–0.24)	0.50
Bristol stool score (1 to 7)	3.6 ± 1.4	0.04 (−0.57–0.64)	4.1 ± 1.2	0.50 (−0.03–1.03)	0.13
Number of bowel motions in previous 24 h	1.6 ± 0.9	0.29 (−0.05–0.62)	1.8 ± 1.0	0.46 (0.08–0.85)	0.70
Patient-reported health score (AQoL-4D)	16.0 (13.0–18.0)	0.25 (−0.65–1.15)	15.0 (13.0–18.5)	0.54 (−0.63–1.70)	0.83

Data are presented as means ± SD, median (25th–75th percentiles), or numbers (%); CI = confidence interval. GSRS, Gastrointestinal Symptom Rating Scale; score > 3 indicates moderate discomfort. ^a^ *p*-values calculated for the group effect using analysis of covariance.

**Table 3:** Serious adverse events by treatment group.

	Placebo (*n* = 28)	Synbiotics (*n* = 28)
Total SAE (*n* = 18)	7 (39%)	11 (61%)
Initial hospitalization	7 (39%)	10 (56%)
Infection, unrelated	2 (11%)	3 (17%)
Cardiovascular event, unrelated	4 (22%)	2 (11%)
Fall, unrelated	1 (6%)	1 (6%)
Surgery, unrelated		4 (22%)
Death, unrelated		1 (6%)

Data are presented as frequency (%). SAE, serious adverse event.

**Table 4:** Exploratory outcomes at end of intervention and changes from baseline.

	Placebo (*n* = 28)	Change (95% CI) Placebo	Synbiotics (*n* = 28)	Change (95% CI) Synbiotics	*p*-Value ^a^
Echocardiographic characteristics					
Global longitudinal strain (%) (*n* = 52)	−17.2 ± 3.5	0.40 (−0.96–1.77)	−17.6 ± 3.2	0.01 (−1.28–1.31)	0.98
Left ventricular mass index (g/m^2^) (*n* = 55)	72.6 (63.2–88.0)	−5.00 (−11.9–1.93)	81.0 (69.0–94.4)	−6.40 (−15.9–3.11)	0.29
Ejection fraction (%) (*n* = 54)	58.0 (53.5–63.0)	−2.86 (−5.82–0.11)	60.5 (57.0–64.0)	−1.23 (−4.61–2.08)	0.25
Uremic toxins (*n* = 55)					
Total indoxyl sulfate (µmol/L)	13.0 (8.0–18.5)	−3.07 (−9.14–2.99)	12.0 (6.7–28.0)	1.50 (−3.25–6.26)	0.96
Total *p*-cresyl sulfate (µmol/L)	64.5 (24.0–135.0)	−17.2 (−49.8–15.3)	69.0 (30.0–179.0)	28.8 (−6.32–64.0)	0.15
Free indoxyl sulfate (µmol/L)	0.8 (0.4–1.2)	−0.09 (−0.34–0.17)	0.8 (0.5–1.2)	0.10 (−0.14–0.34)	0.25
Free *p*-cresyl sulfate (µmol/L)	2.6 (0.9–5.1)	−0.17 (−1.18–0.84)	2.7 (1.4–5.8)	0.98 (0.17–1.79)	0.08
Blood pressure					
Systolic BP (mmHg)	142 ± 17	4.11 (−3.21–11.4)	142 ± 19	1.79 (−6.95–10.5)	0.89
Diastolic BP (mmHg)	81 ± 9	2.39 (−0.81–5.59)	77 ± 8	1.18 (−2.36–4.72)	0.16
eGFR (mL/min/1.73 m^2^)	38.5 (29.5–49.0)	2.61 (−0.41–5.63)	29.0 (20.0–36.0)	−3.14 (−6.23–−0.06)	<0.01
Creatinine (µmol/L)	149 (126–182)	−9.79 (−21.7–2.09)	177 (156–249)	20.8 (2.97–38.5)	<0.01
Cholesterol (mmol/L)	4.6 (3.6–5.0)	−0.07 (−0.37–0.23)	4.1 (3.4–5.0)	−0.07 (−0.34–0.20)	0.59
HDL cholesterol (mmol/L) (*n* = 55)	1.1 (0.9–1.2)	−0.02 (−0.07–0.04)	1.1 (0.9–1.5)	0.05 (−0.23–0.32)	0.57
LDL cholesterol (mmol/L) (*n* = 55)	2.4 (1.7–2.7)	−0.26 (−0.51–−0.01)	2.0 (1.7–2.7)	−0.09 (−0.25–0.08)	0.84
Triglycerides (mmol/L)	1.9 (1.4–2.6)	0.18 (−0.11–0.47)	1.3 (0.9–2.1)	−0.03 (−0.31–0.25)	0.14

Data are presented as means ± SD, median (25th–75th percentiles), or numbers (%); CI = confidence interval. ^a^ *p*-values calculated for the group effect using analysis of covariance.

## Data Availability

Data available upon written request to corresponding author.

## Supplementary Materials

The following are available online at https://www.mdpi.com/article/10.3390/nu13124481/s1, Figure S1: (A) Richness of the gastrointestinal microbiota, by species. (B) Diversity of gastrointestinal microbiota, by species, Figure S2: Redundancy analysis (RDA) of gut microbiome composition by condition, Figure S3: Functional groups of the gastrointestinal microbiota differentiating between participants after placebo or synbiotic supplementation as identified by sparse Partial Least Squares Discriminant Analysis (sPLS-DA), Table S1: Bacterial taxa, by relative abundance at end of intervention.

## Appendix A. Materials and Methods

### Appendix A.1. Evaluation Cardiovascular Structure and Function

Left ventricular systolic function was measured by global longitudinal strain (GLS) using two-dimensional speckle strain. GLS measurements were performed off-line using commercially available dedicated automated software (EchoPAC PC, Version 11, GE Healthcare, Horten, Norway) by experienced observers blinded to clinical and outcome data. Speckles were tracked frame-by-frame throughout the LV wall during the cardiac cycle, and basal, mid, and apical regions of interest were created. GLS was calculated as the mean strain of all 18 segments.

Conventional two-dimensional echocardiography was performed at rest using standard equipment (Vicid & General Electric Medical Systems, Milwaukee, WI, USA) to assess left ventricular measurements as per the guidelines from the American Society of Echocardiography [36]. Left ventricular mass (LVM) was calculated using a standardized formula. Left ventricular mass index was assessed as LVM divided by body surface area or by height. Echocardiography was used to determine E/e’ using a standard formula.

### Appendix A.2. Biochemical Measurement

Following an overnight fast, venous blood samples were collected from all participants. Samples were separated in a refrigerated centrifuge to provide plasma and serum, stored at −80 °C, and analyzed in a single batch. Standard automated laboratory techniques were used to measure serum creatinine. The chronic kidney disease epidemiology calculation (CKD-EPI) calculation in accordance with the Kidney Disease: Improving Global Outcomes (KDIGO) clinical recommendation was used to calculate glomerular filtration rate [37].

### Appendix A.3. Uremic Toxins

Free and protein-bound concentrations of serum IS and PCS were analyzed by Ultra Performance Liquid Chromatography (UPLC) using a fluorescence detection method [38].

### Appendix A.4. Stool Microbiota Analysis

The detailed protocols used for stool collection, DNA extraction, library construction and sequencing, raw data quality checks, filtering to remove host-derived and low-quality microbial reads, and the ensuing data analyses for microbial diversity and functional characterization are all described by McFarlane et al. [20]. All these protocols follow standardized protocols and workflows developed by Microba [39] as part of their standard fee-for-service data generation and analysis.

## References

[1] Weiner D.E. (2004). Chronic Kidney Disease as a Risk Factor for Cardiovascular Disease and All-Cause Mortality: A Pooled Analysis of Community-Based Studies. J. Am. Soc. Nephrol..

[2] Stenvinkel P., Carrero J.J., Axelsson J., Lindholm B., Heimburger O., Massy Z. (2008). Emerging biomarkers for evaluating cardiovascular risk in the chronic kidney disease patient: How do new pieces fit into the uremic puzzle?. Clin. J. Am. Soc. Nephrol..

[3] Moradi H., Sica D.A., Kalantar-Zadeh K. (2013). Cardiovascular burden associated with uremic toxins in patients with chronic kidney disease. Am. J. Nephrol..

[4] Liabeuf S., Barreto D.V., Barreto F.C., Meert N., Glorieux G., Schepers E., Temmar M., Choukroun G., Vanholder R., Massy Z.A. (2010). Free p-cresyl sulphate is a predictor of mortality in patients at different stages of chronic kidney disease. Nephrol. Dial. Transpl..

[5] Lin C.J., Liu H.L., Pan C.F., Chuang C.K., Jayakumar T., Wang T.J., Chen H.H., Wu C.J. (2012). Indoxyl sulfate predicts cardiovascular disease and renal function deterioration in advanced chronic kidney disease. Arch. Med. Res..

[6] Snelson M., Biruete A., McFarlane C., Campbell K. (2020). A Renal Clinician’s Guide to the Gut Microbiota. J. Ren. Nutr..

[7] Meijers B.K., Evenepoel P. (2011). The gut-kidney axis: Indoxyl sulfate, p-cresyl sulfate and CKD progression. Nephrol. Dial Transpl..

[8] Rossi M., Johnson D.W., Morrison M., Pascoe E.M., Coombes J.S., Forbes J.M., Szeto C.C., McWhinney B.C., Ungerer J.P., Campbell K.L. (2016). Synbiotics Easing Renal Failure by Improving Gut Microbiology(SYNERGY): A Randomized Trial. Clin. J. Am. Soc. Nephrol..

[9] McFarlane C., Ramos C.I., Johnson D.W., Campbell K.L. (2019). Prebiotic, Probiotic, and Synbiotic Supplementation in Chronic Kidney Disease: A Systematic Review and Meta-analysis. J. Ren. Nutr..

[10] MacLaughlin H.L., Sarafidis P.A., Greenwood S.A., Campbell K.L., Hall W.L., Macdougall I.C. (2012). Compliance With a Structured Weight Loss Program Is Associated With Reduced Systolic Blood Pressure in Obese Patients With Chronic Kidney Disease. Am. J. Hypertens..

[11] Cedillo-Couvert E.A., Ricardo A.C., Chen J., Cohan J., Fischer M.J., Krousel-Wood M., Kusek J.W., Lederer S., Lustigova E., Ojo A. (2018). Self-reported Medication Adherence and CKD Progression. Kidney Int. Rep..

[12] Ash S., Campbell K., MacLaughlin H., McCoy E., Chan M., Anderson K., Corke K., Dumont R., Lloyd L., Meade A. (2006). Evidence based practice guidelines for the nutritional management of chronic kidney disease. Nutr. Diet.

[13] Chan M.J.D. Modification of Lifestyle and Nutrition Interventions for Management of Early Chronic Kidney Disease. http://www.cari.org.au/CKD/CKD%20early/.

[14] Fuentes-Zaragoza E., Sánchez-Zapata E., Sendra E., Sayas E., Navarro C., Fernández-López J., Pérez-Alvarez J.A. (2011). Resistant starch as prebiotic: A review. Starch–Stärke.

[15] Gibson G.R.W.X. (1994). Regulatory effects of bifidobacteria on the growth of other colonic bacteria. J. Appl. Bacteriol..

[16] Evenepoel P., Meijers B.K., Bammens B.R., Verbeke K. (2009). Uremic toxins originating from colonic microbial metabolism. Kidney Int. Suppl..

[17] Svedlund J., Sjödin I., Dotevall G. (1988). GSRS—A clinical rating scale for gastrointestinal symptoms in patients with irritable bowel syndrome and peptic ulcer disease. Dig. Dis. Sci..

[18] O’Donnell L.J.V.J., Heaton K.W. (1990). Detection of pseudodiarrhoea by simple clinical assessment of intestinal transit rate. BMJ.

[19] Hawthorne G., Richardson J., Osborne R. (1999). The Assessment of Quality of Life (AQoL) instrument: A psychometric measure of Health-Related Quality of Life. Qual. Life Res..

[20] Mcfarlane C., Krishnasamy R., Stanton T., Savill E., Snelson M., Mihala G., Morrison M., Johnson D.W., Campbell K.L. (2021). Diet Quality and Protein-Bound Uraemic Toxins: Investigation of Novel Risk Factors and the Role of Microbiome in Chronic Kidney Disease. J. Ren. Nutr..

[21] Buttigieg P.L., Ramette A. (2014). A guide to statistical analysis in microbial ecology: A community-focused, living review of multivariate data analyses. FEMS Microbiol. Ecol..

[22] Le Cao K.A., Martin P.G., Robert-Granie C., Besse P. (2009). Sparse canonical methods for biological data integration: Application to a cross-platform study. BMC Bioinform..

[23] Dehghani H.H.F., Mozaffari-Khosravi H., Nouri-Majelan N., Dehghani A. (2016). Synbiotic Supplementations for Azotemia in Patients With Chronic Kidney Disease: A Randomized Controlled Trial. Iran J. Kidney Dis..

[24] Kooshki A., Tofighiyan T., Miri M. (2019). A synbiotic supplement for inflammation and oxidative stress and lipid abnormalities in hemodialysis patients. Hemodial Int..

[25] Soleimani A., Motamedzadeh A., Zarrati Mojarrad M., Bahmani F., Amirani E., Ostadmohammadi V., Tajabadi-Ebrahimi M., Asemi Z. (2019). The Effects of Synbiotic Supplementation on Metabolic Status in Diabetic Patients Undergoing Hemodialysis: A Randomized, Double-Blinded, Placebo-Controlled Trial. Probiotics Antimicrob. Proteins.

[26] Viramontes-Horner D., Marquez-Sandoval F., Martin-del-Campo F., Vizmanos-Lamotte B., Sandoval-Rodriguez A., Armendariz-Borunda J., Garcia-Bejarano H., Renoirte-Lopez K., Garcia-Garcia G. (2015). Effect of a symbiotic gel (*Lactobacillus acidophilus* + *Bifidobacterium lactis* + inulin) on presence and severity of gastrointestinal symptoms in hemodialysis patients. J. Ren. Nutr..

[27] March D.S., Jones A.W., Bishop N.C., Burton J.O. (2020). The Efficacy of Prebiotic, Probiotic, and Synbiotic Supplementation in Modulating Gut-Derived Circulatory Particles Associated With Cardiovascular Disease in Individuals Receiving Dialysis: A Systematic Review and Meta-analysis of Randomized Controlled Trials. J. Ren. Nutr..

[28] Firouzi S., Haghighatdoost F. (2018). The effects of prebiotic, probiotic, and synbiotic supplementation on blood parameters of renal function: A systematic review and meta-analysis of clinical trials. Nutrition.

[29] O’Callaghan A., van Sinderen D. (2016). Bifidobacteria and Their Role as Members of the Human Gut Microbiota. Front. Microbiol..

[30] Liu X., Mao B., Gu J., Wu J., Cui S., Wang G., Zhao J., Zhang H., Chen W. (2021). Blautia-a new functional genus with potential probiotic properties?. Gut Microbes.

[31] Vital M., Howe A.C., Tiedje J.M. (2014). Revealing the bacterial butyrate synthesis pathways by analyzing (meta)genomic data. mBio.

[32] den Besten G., van Eunen K., Groen A.K., Venema K., Reijngoud D.J., Bakker B.M. (2013). The role of short-chain fatty acids in the interplay between diet, gut microbiota, and host energy metabolism. J. Lipid Res..

[33] Ramezani A., Massy Z.A., Meijers B., Evenepoel P., Vanholder R., Raj D.S. (2016). Role of the Gut Microbiome in Uremia: A Potential Therapeutic Target. Am. J. Kidney Dis..

[34] Cruz-Mora J., Martinez-Hernandez N.E., Martin del Campo-Lopez F., Viramontes-Horner D., Vizmanos-Lamotte B., Munoz-Valle J.F., Garcia-Garcia G., Parra-Rojas I., Castro-Alarcon N. (2014). Effects of a symbiotic on gut microbiota in Mexican patients with end-stage renal disease. J. Ren. Nutr..

[35] Guida B., Germano R., Trio R., Russo D., Memoli B., Grumetto L., Barbato F., Cataldi M. (2014). Effect of short-term synbiotic treatment on plasma p-cresol levels in patients with chronic renal failure: A randomized clinical trial. Nutr. Metab. Cardiovasc. Dis..

[36] Lang R.M., Badano L.P., Mor-Avi V., Afilalo J., Armstrong A., Ernande L., Flachskampf F.A., Foster E., Goldstein S.A., Kuznetsova T. (2015). Recommendations for cardiac chamber quantification by echocardiography in adults: An update from the American Society of Echocardiography and the European Association of Cardiovascular Imaging. J. Am. Soc. Echocardiogr..

[37] Kidney Disease: Improving Global Outcomes (KDIGO) CKD Work Group (2013). KDIGO 2012 Clinical Practice Guideline for the Evaluation and Management of Chronic Kidney Disease. Kidney Int. Suppl..

[38] Pretorius C.J., McWhinney B.C., Sipinkoski B., Johnson L.A., Rossi M., Campbell K.L., Ungerer J.P. (2013). Reference ranges and biological variation of free and total serum indoxyl- and p-cresyl sulphate measured with a rapid UPLC fluorescence detection method. Clin. Chim. Acta.

[39] Parks D.H., Rigato F., Krause L., Hugenholtz P., Tyson G.W., Wood D.L. Evaluation of the Microba Community Profiler (MCP) for Taxonomic Profiling of Metagenomic Datasets from the Human Gut Microbiome. http://insight.microba.com.

