# Supplementary material for: Synbiotics Easing Renal Failure by Improving Gut Microbiology II (SYNERGY II): A Feasibility Randomized Controlled Trial
Source: Nutrients. 2021 Dec 15;13(12):4481. doi: 10.3390/nu13124481 (PMC8708915; doi:10.3390/nu13124481)

Supplemental Material

Item S1 Supplemental Tables -2-

Item S2 Supplemental Figures -3-

Item S1: Supplemental Tables

Supplemental Table S1: Bacterial taxa, by relative abundance at end of intervention.

Taxonomy	Abundance Placebo (mean ± SD)	Abundance Synbiotics (mean ± SD)	Fold Change	FDR	P-value (clr)
**Phylum**					
Actinobacteria	4.6 ± 5.1	3.3 ± 3.5	-1.4	0.061	0.011
Euryarchaeota	0.097 ± 0.25	0.086 ± 0.16	-1.1	0.061	0.0079
**Family**					
Streptococcaceae	1.3 ± 2.8	0.3 ± 0.34	-4.3	0.065	0.0014
Methanobacteriaceae	0.097 ± 0.25	0.086 ± 0.16	-1.1	0.11	0.0049
Bifidobacteriaceae	2.3 ± 4.0	2.5 ± 3.3	1.1	0.21	0.014
CAG-272	0.04 ± 0.091	0.024 ± 0.06	-1.7	0.41	0.037
**Genus**					
Streptococcus	1.3 ± 2.8	0.3 ± 0.34	-4.3	0.20	0.0015
Methanobrevibacter_A	0.097 ± 0.25	0.086 ± 0.16	-1.1	0.32	0.006
Ruminiclostridium_C	0.35 ± 0.5	0.21 ± 0.26	-1.7	0.32	0.0073
Bifidobacterium	2.3 ± 4.0	2.5 ± 3.3	1.1	0.43	0.013
Eubacterium_G	0.033 ± 0.064	0.034 ± 0.068	1	0.56	0.022
CAG-226	0.1 ± 0.26	0.11 ± 0.32	1.1	0.56	0.034
Intestinimonas	0.022 ± 0.079	0.059 ± 0.15	2.7	0.56	0.038
**Species**					
s__Bifidobacterium animalis	0.55 ± 2.7	1.3 ± 2.8	2.4	0.04	0.00017
s__Bacteroides cellulosilyticus	0.49 ± 1.6	0.28 ± 1.1	-1.7	0.47	0.0039
s__Blautia MIC1901	0.039 ± 0.12	0.093 ± 0.19	2.4	0.47	0.0084
s__Ruminiclostridium_C MIC856	0.35 ± 0.5	0.2 ± 0.27	-1.7	0.47	0.009
s__Duodenibacillus MIC4494	0.22 ± 1.1	0.24 ± 0.74	1.1	0.47	0.013
s__Streptococcus thermophilus	0.21 ± 0.89	0.22 ± 0.33	1	0.47	0.015
s__Subdoligranulum MIC5784	0.055 ± 0.14	0.13 ± 0.23	2.4	0.47	0.018
s__Alistipes senegalensis	0.014 ± 0.039	0.011 ± 0.036	-1.3	0.47	0.019
s__Acidaminococcus intestini	0.039 ± 0.2	0.034 ± 0.1	-1.1	0.47	0.019
s__Eubacterium_E hallii	0.38 ± 0.5	0.095 ± 0.18	-4	0.47	0.023
s__Bacteroides_B vulgatus	1.7 ± 2.6	3.5 ± 4.0	2.1	0.47	0.024
s__Prevotella MIC849	0.55 ± 1.0	0.071 ± 0.21	-7.7	0.47	0.026
s__CAG-312 MIC3688	0.0056 ± 0.021	0.028 ± 0.097	5	0.47	0.026
s__Methanobrevibacter_A smithii	0.06 ± 0.17	0.071 ± 0.15	1.2	0.47	0.028
s__CAG-226 MIC3942	0.1 ± 0.26	0.11 ± 0.32	1.1	0.53	0.036
s__Alistipes MIC3765	0.038 ± 0.087	0.24 ± 0.69	6.3	0.53	0.036
s__Butyricimonas MIC1139	0.0023 ± 0.013	0.013 ± 0.028	5.7	0.53	0.042
s__CAG-103 MIC5000	0.25 ± 0.61	0.11 ± 0.35	-2.3	0.53	0.044
s__Ruthenibacterium lactatiformans	0.85 ± 2.0	0.065 ± 0.19	-13	0.53	0.049
s__CAG-103 MIC465	0.0073 ± 0.04	0.075 ± 0.14	10	0.53	0.049

Item S2: Supplemental Figure Legends

Supplemental Figure S1:	A) Richness of the gastrointestinal microbiota, by species.
	B) Diversity of gastrointestinal microbiota, by species.
Supplemental Figure S2:	Redundancy analysis (RDA) of gut microbiome composition by condition.
Supplemental Figure S3:	Functional groups of the gastrointestinal microbiota differentiating between participants after placebo or synbiotic supplementation as identified by sparse Partial Least Squares Discriminant Analysis (sPLS-DA).

Supplemental Figure S1A

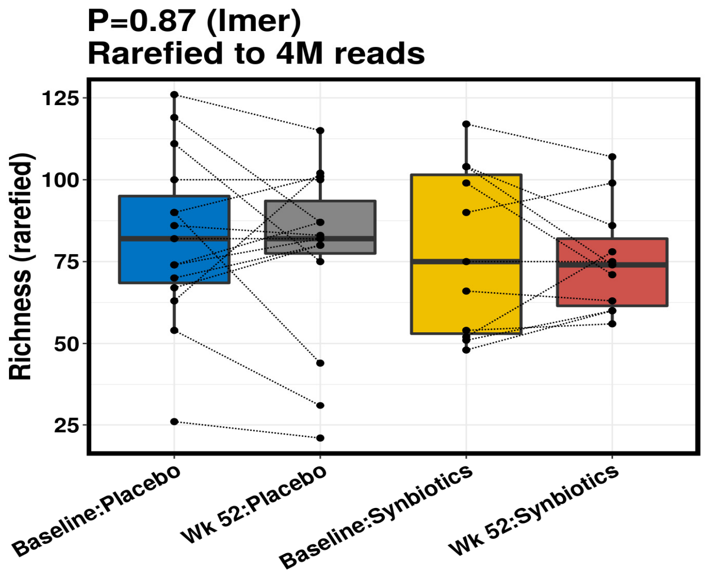

Supplemental Figure S1B

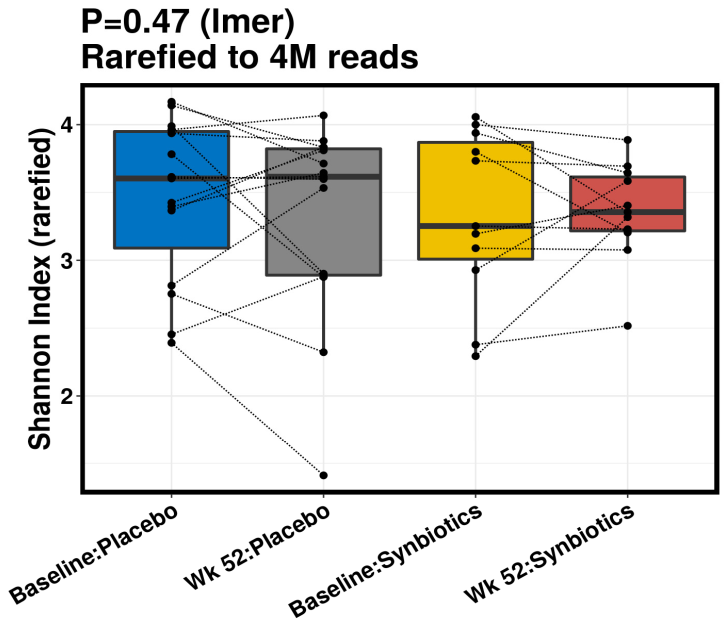

Supplemental Figure S2

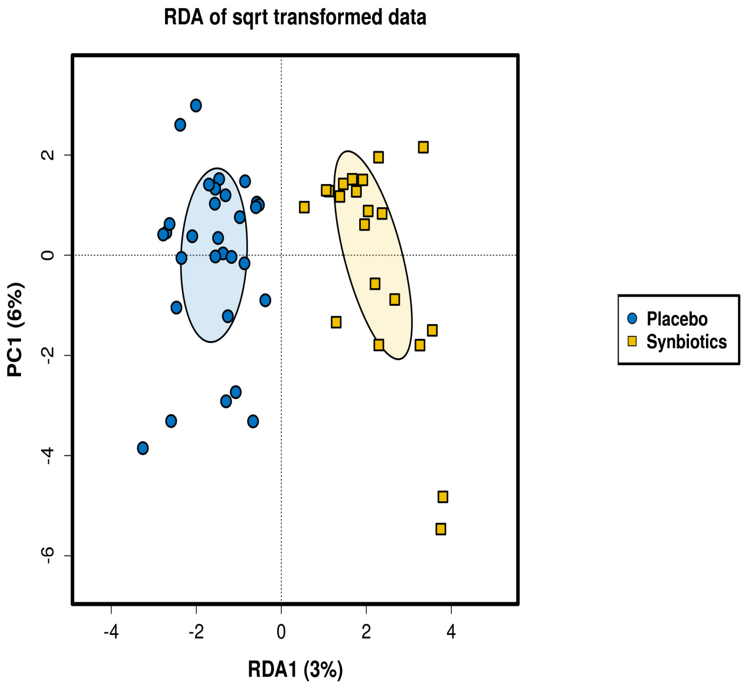

Supplemental Figure S3

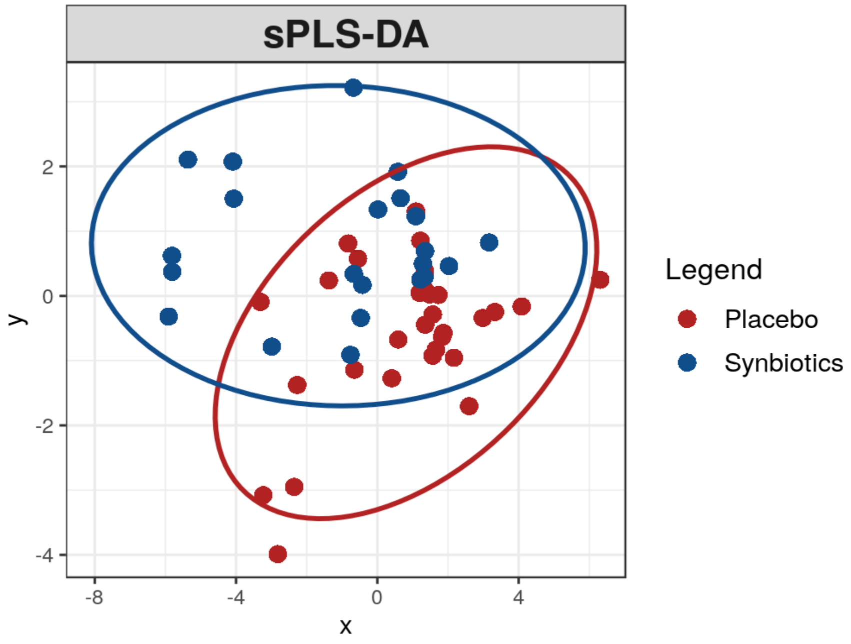

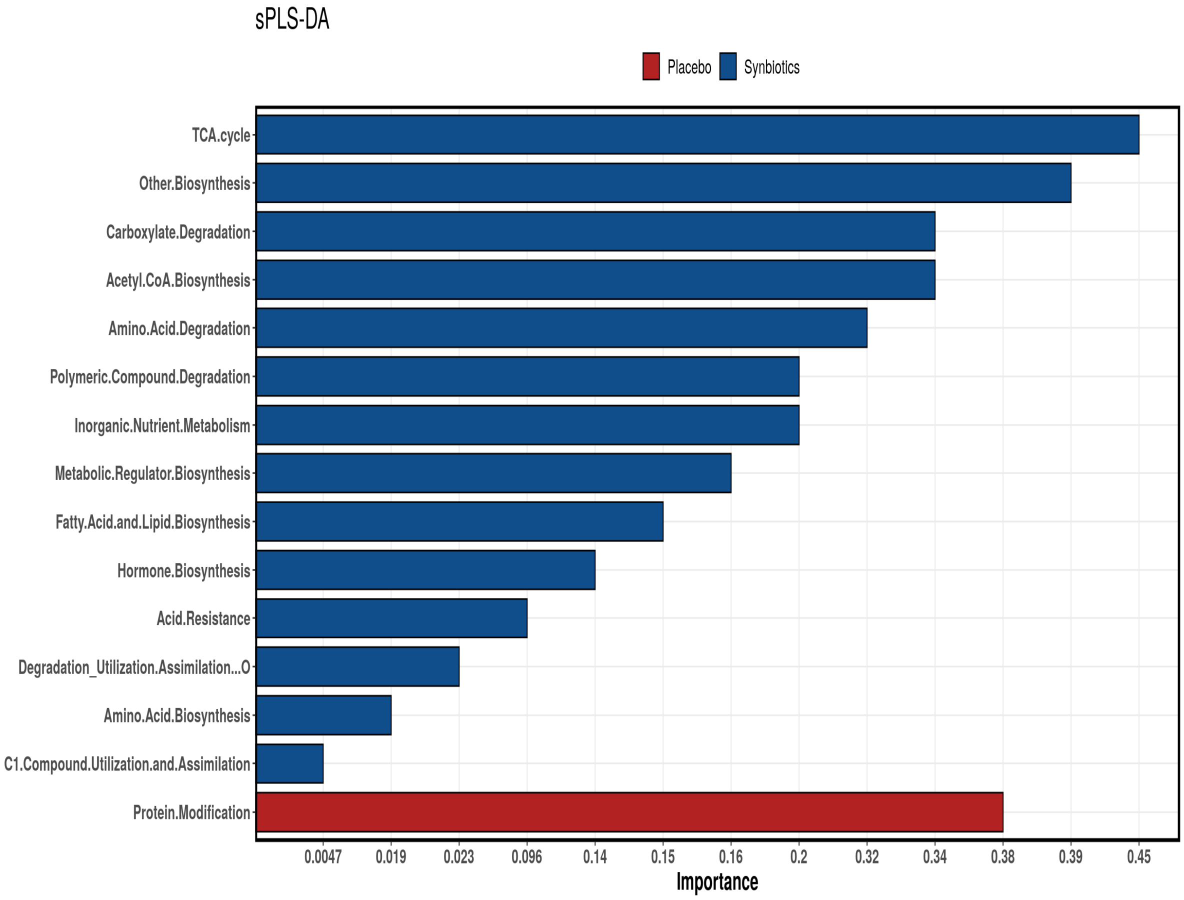

Supplement: Supplementary file 1 [file nutrients-13-04481-s001.zip › nutrients-1502290-supplementary.pdf]
